# Anti-Inflammatory and Immunomodulatory Effects of the *Grifola frondosa* Natural Compound *o*-Orsellinaldehyde on LPS-Challenged Murine Primary Glial Cells. Roles of NF-κβ and MAPK

**DOI:** 10.3390/pharmaceutics13060806

**Published:** 2021-05-28

**Authors:** Sarah Tomas-Hernandez, Jordi Blanco, Santiago Garcia-Vallvé, Gerard Pujadas, María José Ojeda-Montes, Aleix Gimeno, Lluís Arola, Luisa Minghetti, Raúl Beltrán-Debón, Miquel Mulero

**Affiliations:** 1 Cheminformatics and Nutrition Group, Department of Biochemistry and Biotechnology, Campus Sescelades, Universitat Rovira i Virgili (URV), 43007 Tarragona, Catalonia, Spain; sarahtomas89@gmail.com (S.T.-H.); santi.garcia-vallve@urv.cat (S.G.-V.); gerard.pujadas@urv.cat (G.P.); Maria.Ojedamontes@sib.swiss (M.J.O.-M.); aleix.gimeno@irbbarcelona.org (A.G.); 2 Physiology Unit, Laboratory of Toxicology and Environmental Health, Research in Neurobehavior and Health (NEUROLAB), School of Medicine, IISPV, Universitat Rovira i Virgili (URV), 43202 Tarragona, Catalonia, Spain; jordi.blanco@urv.cat; 3 Molecular Modeling Group, SIB Swiss Institute of Bioinformatics, 1015 Lausanne, Switzerland; 4 Joint IRB-BSC-CRG Program in Computational Biology, Institute for Research in Biomedicine (IRB Barcelona), Baldiri Reixac 10-12, 08020 Barcelona, Catalonia, Spain; 5 Nutrigenomics Research Group, Department of Biochemistry and Biotechnology, Campus Sescelades, Universitat Rovira i Virgili (URV), 43007 Tarragona, Catalonia, Spain; lluis.arola@urv.cat; 6 Department of Cell Biology and Neurosciences, Istituto Superiore di Sanità, 00161 Rome, Italy; luisa.minghetti@iss.it; 7 MoBioFood Research Group, Departament de Bioquímica i Biotecnologia, Universitat Rovira i Virgili, 43007 Tarragona, Catalonia, Spain; raul.beltran@urv.cat

**Keywords:** microglia, astrocytes, neuro-inflammation, immunomodulation, *Grifola frondosa*, natural compounds, JNK, p38 kinase, *o*-orsellinaldehyde, NF-κB

## Abstract

In response to foreign or endogenous stimuli, both microglia and astrocytes adopt an activated phenotype that promotes the release of pro-inflammatory mediators. This inflammatory mechanism, known as neuroinflammation, is essential in the defense against foreign invasion and in normal tissue repair; nevertheless, when constantly activated, this process can become detrimental through the release of neurotoxic factors that amplify underlying disease. In consequence, this study presents the anti-inflammatory and immunomodulatory properties of *o*-orsellinaldehyde, a natural compound found by an in silico approach in the *Grifola frondosa* mushroom, in astrocytes and microglia cells. For this purpose, primary microglia and astrocytes were isolated from mice brain and cultured in vitro. Subsequently, cells were exposed to LPS in the absence or presence of increasing concentrations of this natural compound. Specifically, the results shown that *o*-orsellinaldehyde strongly inhibits the LPS-induced inflammatory response in astrocytes and microglia by decreasing nitrite formation and downregulating iNOS and HO-1 expression. Furthermore, in microglia cells *o*-orsellinaldehyde inhibits NF-κB activation; and potently counteracts LPS-mediated p38 kinase and JNK phosphorylation (MAPK). In this regard, *o*-orsellinaldehyde treatment also induces a significant cell immunomodulation by repolarizing microglia toward the M2 anti-inflammatory phenotype. Altogether, these results could partially explain the reported beneficial effects of *G. frondosa* extracts on inflammatory conditions.

## 1. Introduction

The participation of the inflammatory process is being increasingly recognized to play detrimental roles in diverse neurological conditions and pathologies including not only primary degenerative disorders such as Parkinson’s and Alzheimer’s diseases but also primary inflammatory diseases such as multiple sclerosis and HIV dementia, as well as in brain injuries as consequence of trauma and stroke [1,2]. Such an inflammation reaction is favorized by the activity of supporting glial cells such as astrocytes and microglia that surrounds the neurons [3].

Microglial cells are the resident macrophages of the nervous system and play very important roles in immune regulation and neuronal homeostasis. Under normal conditions microglia show a ramified morphology and display a very active and continuous surveillance function [4,5]. The activation of microglia is a protective mechanism designed to repair damaged tissues in the early stages of neurodegeneration processes [6]. However, the long-term or excessive activation of these cells is directly correlated with chronic neuroinflammation which will become detrimental due to the overproduction of neurotoxins and cytokines, that could promote a cascade of events that will culminate in a progressive neuronal death [7,8]. In this activation process microglial cells adopt diverse functional phenotypes that range from the alternative anti-inflammatory M2 phenotype, to the classic pro-inflammatory and neurotoxic phenotype, known as M1 [9,10].

Other prevalent glial cell types in the mammalian brain are astrocytes [11]. Interestingly, during the last few decades it has been shown that besides their housekeeping function astrocytes carry out several roles in control and maintenance of brain health [12]. After a brain insult, astrocytes undergo a process known as “reactive astrogliosis” [13]. Under this condition, astrocytes suffer significant morphological changes along with significant gene expression alterations of chemokines and pro-inflammatory cytokines, among other transcripts [14,15], they also become hypertrophic and increase the expression of glial fibrillary acidic protein [16].

The blood–brain barrier (BBB) is a semipermeable border of endothelial cells which has a highly selective permeability. The BBB restricts the access of molecules, because of: (a) the negative surface polarity; (b) the lack of fenestrations; (c) the tight junctions formed by lateral transmembrane proteins; and (d) the high level of efflux transporters, especially P-glycoprotein (P-gp) [17]. In consequence, the BBB prevents the non-selectively crossing of solutes from the circulating blood into the extracellular fluid of the central nervous system (CNS) where neurons reside. In this regard, for being effective in the brain, drugs must first cross the BBB. The tight junctions form a physical barrier by reducing the permeation of ions and other small hydrophilic solutes through the intercellular cleft (paracellular pathway). In consequence, the essential molecular fluxes must use predominantly transcellular pathways. Nevertheless, it is assumed that the most important permeability process through the BBB is passive diffusion of small compounds, although active transport of compounds may be more significant than originally thought [18]. Although the BBB is protective in nature, the inability for drug molecules to permeate the BBB is a significant impediment for CNS drug candidates and should be addressed early in the drug discovery process [19].

Natural products are considered as a continuous source of bioactive compounds for treating several important chronic human pathologies [20]. More concisely, in the neuropathology field, several neuro health-promoting molecules with interesting properties (i.e., improved synaptic plasticity, neuro-regenerative, neurite-outgrowth, anti-apoptotic, Aβ reduction, AChE and BACE1 inhibition) have been identified in different mushroom species [21]. Additionally, increasing number of fungal species have demonstrated anti-neuroinflammatory properties in the CNS, which has become a growing area for the therapeutic intervention of neurodegenerative diseases [8,22].

Nevertheless, despite the potential value of mushrooms as source of novel biomolecules; the discovery process has been almost restricted to the treatment of cells and/or animals with mushroom-derived extracts enriched on secondary metabolites and/or glycoproteins and polysaccharides (mostly β-glucans) [23].

Virtual screening (VS) has been defined as a computational strategy used in drug discovery to search libraries of small molecules to identify those chemical structures which are most likely to bind to a drug target, typically an enzyme or protein receptor. In consequence, the VS approach represents an additional strategy to further explore and identify new mushroom-derived biomolecules with interesting health benefits. For this purpose, a relevant biological target must be selected which defines the bioactivity outcome. For example, the ubiquitin ligase Mdm2 that controls p53 proteasome degradation has been used for the screening and the discovery of mushroom derived molecules with anti-cancer applications [24]. Previous in silico experiments of our group have identified that the natural compound *o*-orsellinaldehyde contained in the *Grifola* *frondosa* mushroom was a potent inhibitor of the Ikβα kinase. Further in vitro and in vivo experiments evidenced that this compound was able to abrogate systemic inflammation [25]. We wondered if this mushroom-derived compound could also exert its anti-inflammatory activity in the CNS, and if this activity was only restricted to its main target or could have alternative mechanisms (e.g., p38 kinase, JNK), as well as its effect on microglia polarization.

## 2. Materials and Methods

### 2.1. Prediction of Blood–Brain Barrier Permeation

The SwissADME website [26]. was used to compute physicochemical descriptors and predict ADME parameters for the *o*-orsellinaldehyde. The BOILED-Egg method was used to predict blood–brain barrier (BBB) permeation [27]. This method uses a representation of the predicted *n*-octanol/water partition coefficient (WLOGP) v. topological polar surface area (tPSA) to predict the BBB permeation.

### 2.2. Chemicals

DMEM, FBS, Penicillin/Streptomycin (P/S) and L-glutamine were bought from Lonza, Cultek (Barcelona, Spain). L-15 media was obtained from Gibco, Fisher (Madrid, Spain). Protease and Phosphatase inhibitor mixture ((phenymethanesulfonyl fluoride solution (ref: 93482), protease inhibitor cocktail (ref: P8340), phosphatase inhibitor cocktail-2 (ref: P5726), and phosphatase inhibitor cocktail-3 (ref: P0044)), *o*-orsellinaldehyde (ref: 657603), N-1-naftiletilendiamine (NED; ref: 222488), Lipopolisacharide (LPS) from *Escherichia coli* O111:B4 (L5293) all from Sigma-Aldrich Chemical, Madrid, Spain. Antibodies: polyclonal antibody for iNOS (sc-651, Santa Cruz Biochemicals, Dallas, TX, USA), polyclonal antibody for HO-1 (70081; Cell Signaling Technology, Beverly, LA, USA), polyclonal antibody for Phospho-Ikβα (Ser32) (2859; Cell Signaling Technology), polyclonal antibody for Phospho-SAPK/JNK (Thr183/Tyr185) (9251; Cell Signaling Technology), polyclonal antibody for Phospho-p38 MAP Kinase (Thr180/Tyr182) (9211; Cell Signaling Technology), and polyclonal antibody for β-actin (A 2066; Sigma-Aldrich Chemical, San Luis, MO, USA). Anti-rabbit secondary antibody (NA934, Merck, Barcelona, Spain).

### 2.3. Animals and In Vitro Cell Culture Procedure

#### 2.3.1. Acquisition of Glial Cells

All animal care and experimental protocols with animals were approved by the Ethics Review Committee for Animal Experimentation of the Universitat Rovira i Virgili (reference number 4249 by Generalitat de Catalunya; 18/09/19) and were carried out in accordance with Directive 86/609EEC of the Council of the European Union and the procedure established by the Departament d’Agricultura, Ramaderia i Pesca of the Generalitat de Catalunya. 

Mixed glial cell cultures were prepared from the cerebral cortex of three-day-old rats following the protocol described by Tamashiro et al., 2012 [28] with some modifications. Briefly, after animal sacrifice whole brains were rapidly dissected out and placed in a Petri dish with L-15 media on ice (Leibovitz’s medium supplemented with 0.1% BSA and 1% penicillin/streptomycin (P/S)). After the removal of the meninges, the cortices were fragmented and mechanically dissociated. The material was then dispensed through a cell strainer (100 μm pores) and centrifuged. Dissociated cells were seeded onto 75 cm^2^ flasks in Dulbecco’s modified Eagle’s medium supplemented with 10% heat-inactivated fetal bovine serum (FBS) and 1% P/S and the cultures were maintained in a humidified atmosphere of 5% CO_2_ at 37 °C. The medium was replaced on the fifth day after the initial seeding and changed every third day afterwards.

#### 2.3.2. Isolation and Plating of Primary Microglia

Upon reaching confluence (10–12 days), the microglial cells were harvested by mild shaking (4 rcf for 4 h at 37 °C) and put into 50 mL conical tubes. The flasks with the remaining mixed glia were returned to the incubator after adding fresh media. The isolated microglial cells were centrifuged at 2500 rcf for 5 min, resuspended in microglial plating media (Dulbecco’s modified Eagle’s medium supplemented with 10% heat-inactivated fetal equine serum and 1% P/S) and plated at a density of 500,000 cells/mL. Twelve hours later the cells were ready for treatment. 

#### 2.3.3. Astrocytes Plating

The remaining mixed glia cells, which were mainly astrocytes, were detached from the flasks by mild trypsinization. The cells were pelleted by centrifugation at 2500× *g* for 5 min and then seeded at a density of 500,000 cells/mL in Dulbecco’s modified Eagle’s medium supplemented with 10% heat-inactivated fetal bovine serum and 1% penicillin/streptomycin. The cells were allowed to attach overnight and were treated the day after.

### 2.4. Cellular Toxicity

Microglia and astrocytes were seeded in 96-well plates and cultured in microglia plating media, as described above. For the analysis of cell viability an MTT reduction assay was performed. Microglia cells were treated with different concentrations of *o*-orsellinaldehyde for 24 h. Afterwards, the medium was replaced by 200 µL of fresh plating medium and MTT salt was added at a final concentration of 5 mg/mL. The plate was incubated at 37 °C for 4 h without light. The colored formazan product was then dissolved in DMSO and quantified using a scanning multi-well spectrophotometer (BioTek EON, Izasa, Barcelona, Spain) at a wavelength of 570 nm.

### 2.5. Cell Treatments

#### 2.5.1. Astrocytes Treatment

Astrocytes were seeded into a 12-well plate at a density of 500,000 cells/mL. Twelve hours after plating, the cells were pre-incubated with vehicle (DMSO) or increasing concentrations of *o*-orsellinaldehyde for 30 min and then the inflammatory stimulus was added (LPS 10 ng/mL). As a negative control, some cells were left untreated. Twenty-four hours after the LPS addition, the culture medium was collected for nitrite determination and cells were harvested for further protein analysis by Western blotting.

#### 2.5.2. Microglia Treatment

The microglial cells were treated with vehicle or with the highest concentration tested of *o*-orsellinaldehyde (50 µg/mL) 30 min prior to LPS stimulation (LPS 10 ng/mL). Following 24 h incubation, the medium was collected for NO measurement. Cells were then harvested, and total RNA and protein were isolated for further analysis.

### 2.6. Nitrite Determination

Concentrations of NO in cell culture supernatants of microglia and astrocytes were measured by Griess reaction [29]. Briefly, 50 μL of sample were added to a 96-well microplate and mixed with 100 μL 1% sulfanilamide in 0.5 M HCl. The plate was incubated at 4 °C for 10 min and then 50 μL of NED (N-1-naftiletilendiamine)/well were added. After 30 min at 4 °C the optical density at 540 nm was measured using an ELISA plate reader (BioTek EON, Izasa, Barcelona, Spain). The values were interpolated from a standard curve with known concentrations of nitrite oxide (NO). 

### 2.7. Western Blot Analysis

Cells were washed with PBS and lysed in RIPA buffer (25 mM Tris–HCl; pH 7.4, 150 mM NaCl, 1% NP-40, 1% sodium deoxycholate, 0.1% SDS) containing a protease and phosphatase inhibitor mixture. The lysates were then centrifuged at 11,200rcf for 10 min at 4 °C. The protein concentration was measured using a BCA protein assay (ref: 23225, Thermo/Pierce, Rockford, IL, USA). Equal amounts of protein (30–50 µg) were separated electrophoretically on SDS–polyacrylamide gels and transferred to PVDF membranes (Trans-Blot Turbo system, Bio-Rad, Barcelona, Spain). The membranes were blocked with 5% non-fat milk in PBS–Tween (0.1%) for one hour. They were then blotted overnight at 4 ºC with the appropriate rabbit antibody (dilution 1:1000): polyclonal antibody for iNOS (sc-651, Santa Cruz Biochemicals), polyclonal antibody for HO-1 (70081; Cell Signaling Technology), polyclonal antibody for Phospho-Ikβα (Ser32) (2859; Cell Signaling Technology), polyclonal antibody for Phospho-SAPK/JNK (Thr183/Tyr185) (9251; Cell Signaling Technology), polyclonal antibody for Phospho-p38 MAP Kinase (Thr180/Tyr182) (9211; Cell Signaling Technology) or polyclonal antibody for β-actin (A 2066; Sigma-Aldrich Chemical, San Luis, MO, USA). After washing three times with PBS–Tween, the membranes were hybridized with an anti-rabbit secondary antibody (NA934, Merck, Barcelona, Spain) (dilution 1:10,000) conjugated with horseradish peroxidase for 1 h and then washed three more times with PBS-Tween. The immunoreactive proteins were visualized using an enhanced chemiluminescence substrate kit (ECL Plus; Amersham Biosciences, GE Healthcare, Marlborough, MA, USA) following the manufacturer’s instructions. Digital images were obtained with a GBOX Chemi XL 1.4 system (Syngene, UK), which enables quantification of band intensity. The protein load was monitored via the immuno-detection of β-actin.

### 2.8. RNA Isolation and cDNA Synthesis

Total RNA was obtained from the microglia-treated cells using a RNeasy Mini Kit (QIAgen, Valencia, CA, USA) in accordance with the manufacturer’s protocol. The RNA was resuspended in RNasefree water. A DNase I RNAase free kit (Fermentas, Thermo Scientific, Waltham, MA, USA) was used to remove the genomic DNA from the RNA preparations. The RNA was quantified and tested for purity by measuring absorbance at 260 and 280 nm with a spectrophotometer (Nanodrop 1000 Spectrophotometer, Thermo Scientific, Waltham, MA, USA). Then 1 µg of total RNA from each sample was reverse-transcribed using a First Strand cDNA Synthesis Kit (Fermentas, Thermo Scientific, Waltham, MA, USA) following the supplier’s protocol.

### 2.9. Real-Time RT-PCR

The expression of various genes (iNOS, IL-1β, IL-10, Arg-1 and MRC-1) were evaluated by Q-PCR using a SYBR Premix Ex Taq (Takara, Shiga, Japan) in accordance with the manufacturer’s protocol and analyzed on a CFX96 Real-Time PCR Detection System (BIORAD, Madrid, Spain). Each sample was assessed in triplicate. 

The thermal cycling comprised an initial step at 50 °C for 2 min followed by a polymerase activation step at 95 °C for 10 min and a cycling step with the following conditions: 40 cycles of denaturation at 95 °C for 15 s, annealing at 60 °C for 1 min, and extension at 72 °C for 1 min. At the end of the PCR cycles, the PCR products were analyzed using a heat dissociation protocol to confirm that a single PCR product was detected by the SYBR Green dye. The fluorescence data were acquired at the 72 °C step. The threshold cycle (Ct) was calculated using CFX Manager 3.1 Software (BIORAD, Madrid, Spain) to indicate significant fluorescence signals above the noise during the early cycles of amplification. The software calculated copy numbers for the target samples from the Ct using interpolation from the standard curve.

The relative expression levels of the target genes were calculated relative to GADPH (internal control) in accordance with the 2^−ΔΔCt^ method. The primer sequences for the tested genes are shown in Table 1.

### 2.10. Statistical Analysis

Data are expressed as means ± SD of 3 independent experiments (run in triplicate). Confirmation of normal distribution was carried out by Kolmogorov–Smirnov test. Statistical significance was evaluated using Student’s t test or one-way ANOVA followed by Bonferroni’s correction for multiple comparisons. Significant symbols/letters are indicated in each figure; *p* ≤ 0.05 was accepted as statistical significance. Analyses were performed using GraphPad Prism 8 software (GraphPad Software, San Diego, CA, USA 92108).

## 3. Results

### 3.1. Prediction of Blood–Brain Barrier Permeability

To be effective as a neuroinflammatory modulator, the tested molecule should be able to pass through the BBB to exert its activity on the brain cells. For that reason, as abovementioned, the SwissADME web tool was used to predict the ADME parameters of the *o*-orsellinaldehyde molecule. The BOILED-Egg method was used to predict BBB permeation. As can be seen in Supplementary Figure S1, it was predicted that the molecule would indeed be capable of passing through the blood–brain barrier.

### 3.2. Effects of o-Orsellinaldehyde on Cell Viability of Isolated Astrocytes and Microglia Cells

**Figure 1:**
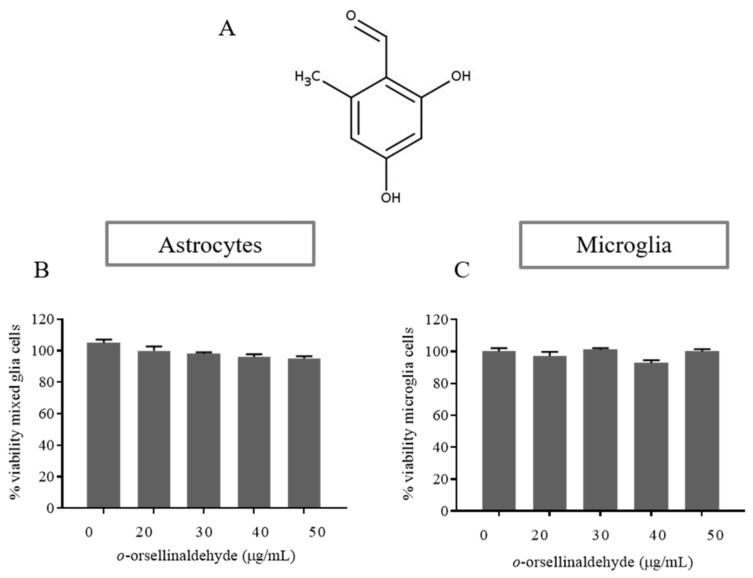
Effect of *o*-orsellinaldehyde on the viability of astrocytes and microglia cells. (**A**) Chemical structure of *o*-orsellinaldehyde. (**B**) MTT results of astrocytes treated with increasing concentrations of *o*-orsellinaldehyde (20–50 µg/mL) for 24 h. (**C**) MTT results of microglia treated with increasing concentrations of *o*-orsellinaldehyde (20–50 µg/mL) for 24 h. Results are shown as the mean ± SD of three independent experiments. Absence of symbols denotes no statistically significant differences.

To determine whether *o*-orsellinaldehyde (depicted in Figure 1A) influences the viability of glial cells, an MTT assay was performed in both isolated astrocytes and microglia cells. The results (Figure 1B,C) demonstrate that *o*-orsellinaldehyde treatment did not affect cell viability after 24 h of incubation (any significant decrease was found among treatments). The concentrations of 20 to 50 µg/mL were therefore selected for further investigation.

**Figure 2:**
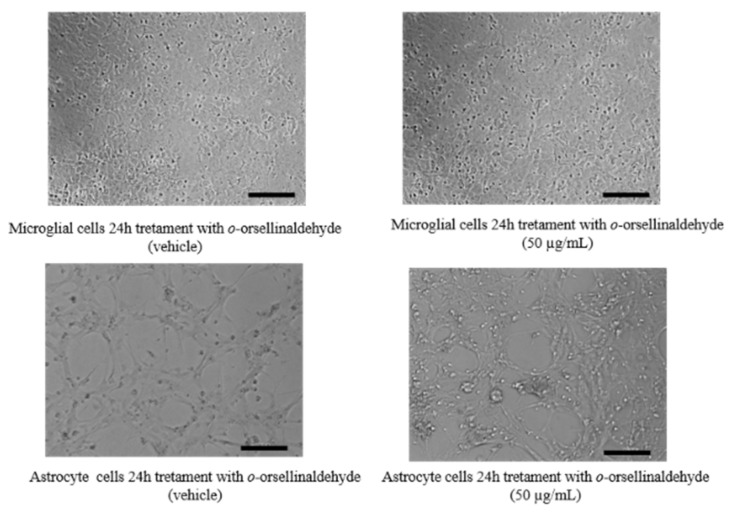
Effect of *o*-orsellinaldehyde on the morphology of astrocytes and microglia cells. Image captions of glial cells (astrocytes and microglia), untreated (**left**) or treated (**right**) with 50 µg/mL of *o*-orsellinaldehyde for 24 h. Image scale bar = 10 µm.

On the other hand, the influence of the treatment on cell morphology was also assessed. No morphological and cytotoxic signals (e.g., cellular shape, membrane damage and floating cells) were observed neither in astrocytes nor in microglial cells (Figure 2).

### 3.3. o-Orsellinaldehyde Inhibits Nitrite Production in LPS-Activated Astrocytes and Microglia Cells

**Figure 3:**
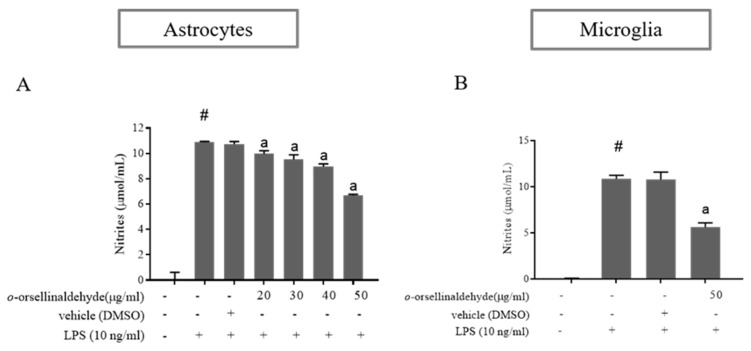
*o*-Orsellinaldehyde inhibits LPS-mediated NO production in astrocytes and microglia cells. Cells were incubated with the indicated concentrations of *o*-orsellinaldehyde for 30 min before treatment, and with LPS (10 ng/mL) for 24 h. The medium was collected for NO measurement by Griess reaction. (**A**) Concentrations of NO in astrocytes. (**B**) Concentrations of NO in microglia. Results are shown as the mean of the nitrite production ± SD of four independent experiments. Statistically significant differences (*p* ≤ 0.05) of LPS compared to the control condition are expressed as a “#”. Statistically significant differences (*p* ≤ 0.05) between LPS conditions are expressed with an “a”.

To evaluate the inhibitory effect of *o*-orsellinaldehyde on NO production, astrocyte cells were pre-incubated with vehicle (DMSO) or increasing concentrations of *o*-orsellinaldehyde 30 min before stimulation with LPS (10 ng/mL). As a negative control, some cells were left untreated. Twenty-four hours after the LPS addition the culture medium was collected, and NO levels were measured by Griess reaction. As shown in Figure 3A, LPS markedly induced nitrite production compared to the control (*p* ≤ 0.05). However, cell pre-treatment with *o*-orsellinaldehyde significantly reduced the levels of NO in the medium in a dose-dependent manner, achieving a reduction of 25–30% with the highest dose of the compound (50 µg/mL). To better characterize the influence of *o*-orsellinaldehyde in other cerebral cell populations, primary microglia cells were isolated from mixed glial cell cultures. The microglial cells were treated with vehicle or with the highest tested concentration of *o*-orsellinaldehyde (50 µg/mL) 30 min prior to stimulation with LPS (10 ng/mL). Following the 24 h incubation, the medium was collected for NO measurement. As Figure 3B shows that *o*-orsellinaldehyde was able to significantly reduce NO production in LPS-stimulated microglia cells. Specifically, it decreased the concentration of NO by 50%.

### 3.4. o-Orsellinaldehyde Decreases iNOS Protein Expression in LPS-Challenged Astrocytes and Microglia Cells

**Figure 4:**
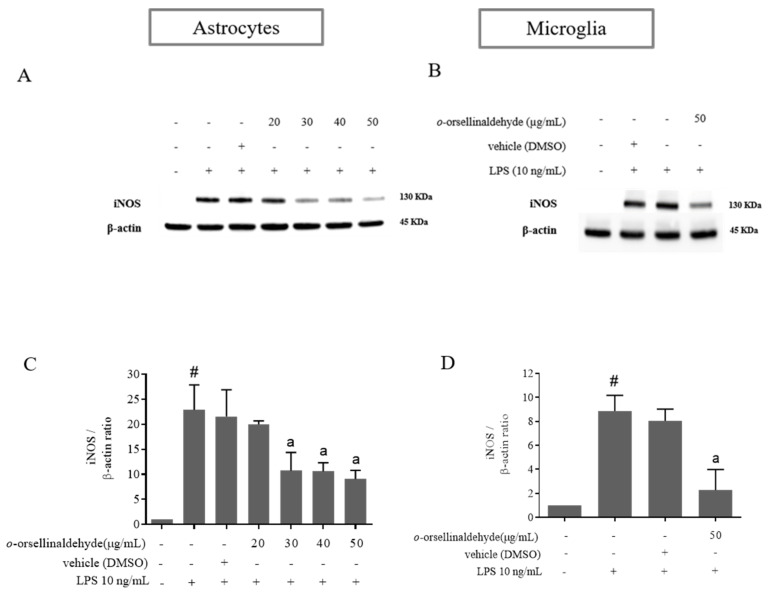
*o*-Orsellinaldehyde downregulates iNOS protein expression in LPS-challenged astrocytes and microglia cells. Cells were incubated with the indicated concentrations of *o*-orsellinaldehyde for 30 min before treatment, and with LPS (10 ng/mL) for 24 h. The cell pellet was collected for iNOS measurement by Western blot. (**A**) Representative image of iNOS Western blotting in astrocytes. (**B**) Representative image of iNOS Western blotting in microglia. (**C**) Densitometry plot of Western blots carried out in astrocytes. (**D**) Densitometry plot of Western blots carried out in astrocytes. Results are shown as the mean ± SD of four independent experiments. Statistically significant differences (*p* ≤ 0.05) of LPS compared to the control condition are expressed as a “#”. Statistically significant differences (*p* ≤ 0.05) between LPS conditions are expressed with an “a”.

To elucidate whether the cause of the NO reduction observed in astrocytes and microglial cells was due to a decrease in iNOS protein levels, iNOS protein expression was determined by immunoblot. As can be seen in Figure 4A–D, LPS increased the expression of this protein in both glial cell populations, while a significant suppression of iNOS protein levels was observed due to the presence of *o*-orsellinaldehyde. In astrocytes the effective dose for iNOS protein downregulation was 0 µg/mL (significant densitometry decrease in comparison to LPS condition). On the other hand, in the case of microglia, although only one dose of the natural compound could be assayed (due to limitations on cell enrichment of microglia primary cultures); it was able to significantly decrease iNOS protein amount (the 50 µg/mL dose reduced nearly 4-fold iNOS expression in comparison to the LPS condition). Consequently, it can be assumed that the decrease in the NO amount was related to iNOS downregulation induced by this natural product.

### 3.5. o-Orsellinaldehyde Decreases HO-1 Protein Expression in LPS-Activated Mixed Glia and Microglia Cells

**Figure 5:**
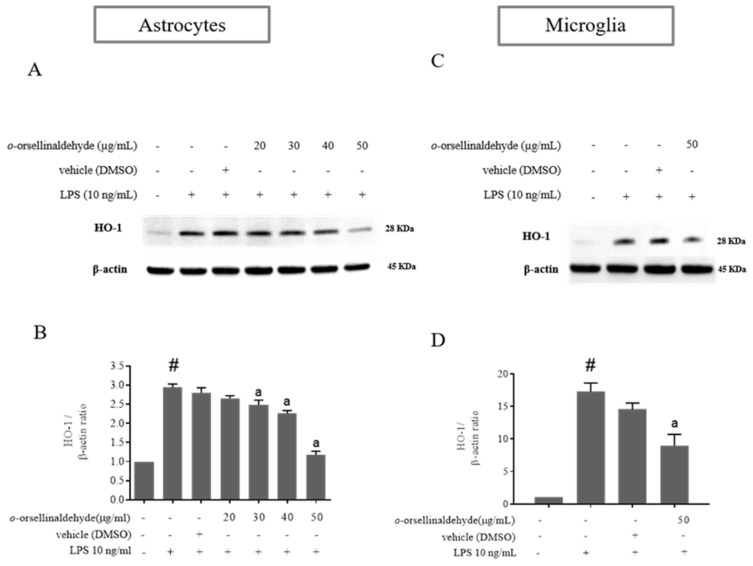
*o*-Orsellinaldehyde counteracts LPS-induced HO-1 protein expression in astrocytes and microglia cells. Cells were incubated with the indicated concentrations of *o*-orsellinaldehyde for 30 min before treatment, and with LPS (10 ng/mL) for 24 h. The cell pellet was collected for HO-1 measurement by Western blot. (**A**) Representative image of iNOS Western blotting in astrocytes. (**B**) Representative image of HO-1 Western blotting in microglia (**C**) Densitometry plot of Western blots carried out in astrocytes. (**D**) Densitometry plot of Western blots carried out in astrocytes. Results are shown as the mean ± SD of four independent experiments. Statistically significant differences (*p* ≤ 0.05) of LPS compared to the control condition are expressed as a “#”. Statistically significant differences (*p* ≤ 0.05) between LPS conditions are expressed with an “a”.

HO-1 expression in monocytes and macrophages has been found to mediate potent anti-inflammatory effects, possibly by restraining them from inducing tissue injury and by modulating their role in the inflammatory response [30]. As shown in Figure 5A–D, LPS-activation of astrocytes and microglia cells notably increased the expression of HO-1 protein. However, astrocyte and microglia pretreatment with *o*-orsellinaldehyde reduced the over-expression of HO-1 caused by the LPS stimulation that could be detrimental for the cells. These results show that the *G. frondosa-* derived natural compound is able to relieve the LPS-stimulated HO-1 expression, which has been described to be mediated by Nrf2 and AREs [31]. Again, as in the iNOS results, regarding the astrocytes the effective dose for HO-1 protein downregulation was 30 µg/mL. Nevertheless, in the case of microglia, although only one dose of the natural compound could be assayed (due to limitations on cell enrichment of microglia primary cultures); it was able to significantly decrease HO-1 protein content (the 50 µg/mL dose reduced nearly 2-fold HO-1 expression in comparison to the LPS condition). Therefore, these results suggest that *o*-orsellinaldehyde is able to counteract the dependence on HO-1 expression of LPS-primmed glial cells, which suggests the protective role of this natural compound.

### 3.6. o-Orsellinaldehyde Suppressed LPS-Induced Microglial Activation Through the Nfkb and MAP Kinase Signaling Pathways

Our previous study [25] indicated that *o*-orsellinaldehyde is able to influence NFkB activation by inhibiting IKβα activity in macrophages. In order to validate that *o*-orsellinaldehyde inhibits IKβα phosphorylation in a neurological context, we examined the NFkB signaling pathway in response to LPS in primary microglial cells. Microglial cells were pre-incubated with *o*-orsellinaldehyde (50 µg/mL) for 30 min, then the LPS (10 ng/mL) was added for 45 more minutes. The cell lysates were subjected to Western blotting for phospho-IKβα. We were also interested in assessing the p38 kinase phosphorylation, and SAPK/JNK phosphorylation.

**Figure 6:**
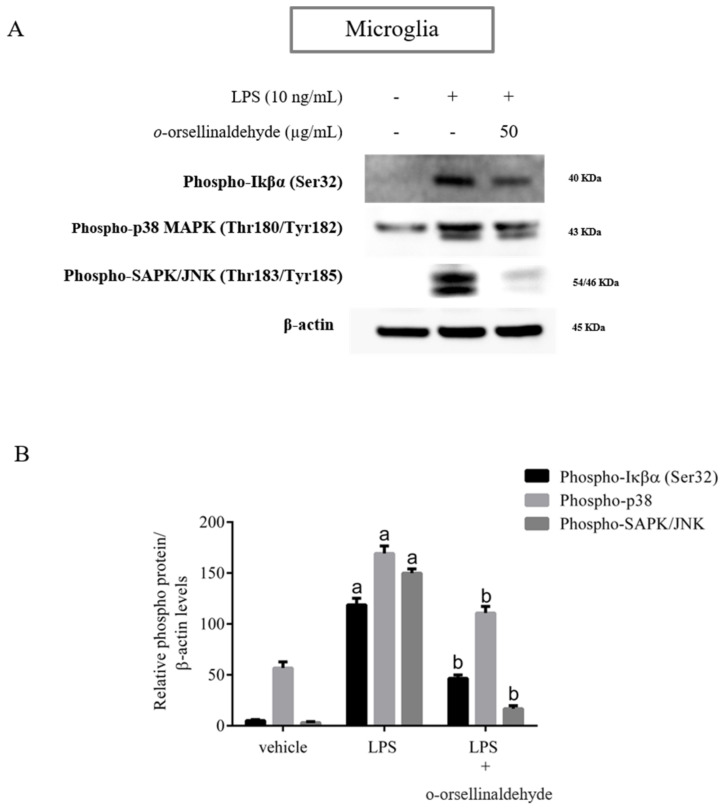
*o*-Orsellinaldehyde abrogates microglial LPS-induced NFκβ and MAPK activation. Cells were incubated with 50 µg/mL of *o*-orsellinaldehyde for 30 min before treatment, and with LPS (10 ng/mL) for 45 min. The cell pellet was collected for phosphorylation measurement by Western blot. (**A**) Representative image of IKβα, p38 and SAPK/JNK phosphorylation levels by Western blotting in microglia. (**B**) Densitometry plot of Western blots carried out in microglia. Results are shown as the mean ± SD of four independent experiments. Statistically significant differences (*p* ≤ 0.05) of LPS compared to the control condition are expressed as an “a”. Statistically significant differences (*p* ≤ 0.05) between LPS conditions are expressed with a “b”.

As Figure 6 depicts, LPS stimulation markedly increased the IKβα phosphorylation. However, *o*-orsellinaldehyde pre-treatment resulted in a strong blockade of this. Regarding the activation levels of p38 and SAPK/JNK kinases, we also observed that the stimulation of microglial cells with LPS lead the cells to a rapid activation of the MAP kinases, whereas pre-incubation of microglia with the tested compound inhibited the phosphorylation of these kinases, showing a total kinase inhibition in the case of SAPK/JNK Kinase. 

These results confirm that the inhibition of the expression of pro-inflammatory mediators by *o*-orsellinaldehyde in LPS-stimulated glia cells is associated with a down-regulation of IKβα phosphorylation due to the IKβα inhibitory capacity of the molecule. Furthermore, the reduced phosphorylation of p38 and SAPK/JNK suggests that the anti-inflammatory effect of *o*-orsellinaldehyde could also be caused by the inhibition of the MAPK signaling pathway.

### 3.7. o-Orsellinaldehyde Alter Microglial M1/M2 Immunological Profile in Response to LPS 

**Figure 7:**
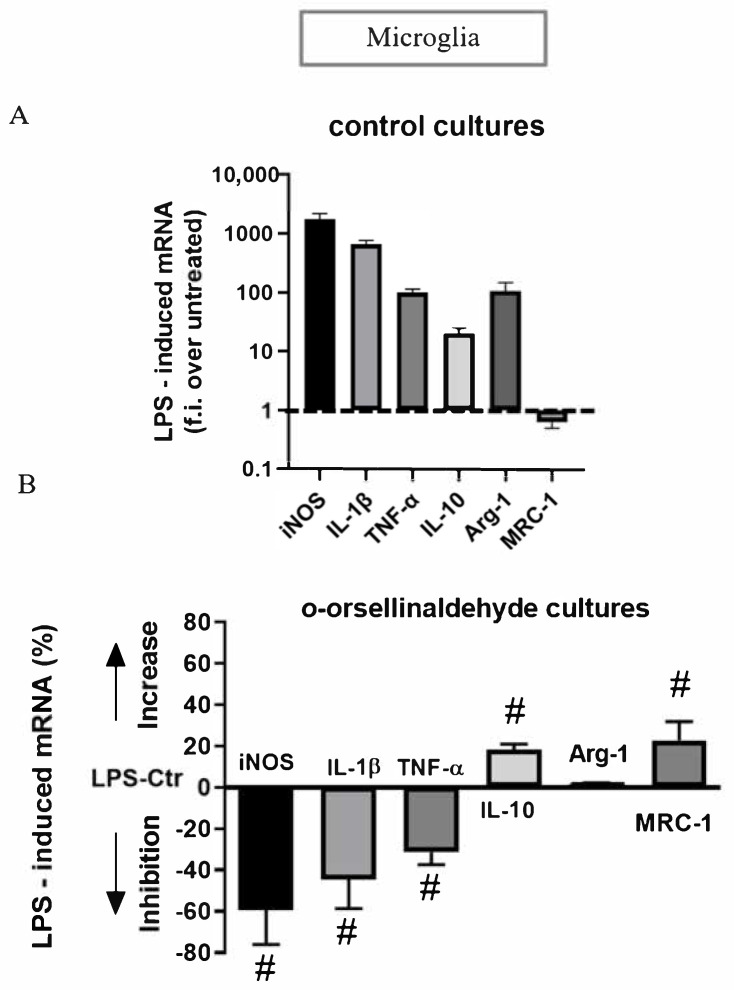
*o*-Orsellinaldehyde induces an immunomodulatory effect in LPS-treated microglial cells. (**A**) Real time PCR analysis of M1 and M2 genes induced by LPS in Ctr microglial cultures. Data are given as folds increase over the expression of the corresponding gene in unstimulated Ctr cultures. Data are mean ± SD of four independent experiments. All values are significantly different from control. (**B**) Real time PCR of M1 and M2 genes induced by LPS in *o*-orsellinaldehyde cultures. Cells were treated and analyzed as described for panel A. Data are given as % of increase or decrease over the corresponding gene in LPS-control medium (Ctr) cultures, with positive values indicating an increase in the expression of the gene in *o*-orsellinaldehyde cultures and negative values an inhibition. Results are shown as mean ± SD of four independent experiments. Symbol “#” denotes significant differences (*p* ≤ 0.05) versus LPS-stimulated Ctr cultures.

Finally, the expression profile of M1 and M2 genes induced by a prototypical inflammatory stimulus like LPS was also assessed. As expected, in control cultures, LPS significantly increased the levels of the iNOS, IL-1β, TNF-α, IL-10, and arginase-1 transcripts and significantly decreased MRC-1 gene expression (Figure 7A). When the LPS-induced mRNA levels in o-orsellinaldehyde cultures were compared to those induced in control cultures, we observed a reduced ability of *o*-orsellinaldehyde microglia treated cells to express iNOS, IL-1β, and TNF-α (Figure 7B). Among the M2 genes, IL-10 and MRC-1 mRNA levels were increased (Figure 7B). However, when looking at Arg-1, a gene whose expression should be increased in M2 as compared to M1 macrophages, mRNA was similar in *o*-orsellinaldehyde as well as in Ctr microglial cells (Figure 7B).

Additionally, although due to technical issues we were no able to take specific photographs, we clearly observed a change in cellular morphology between LPS treated primary microglia (ameboid shape) and LPS + *o*-orsellinaldehyde treated cells (ramified/stellate shape). This further reinforces the concept of the repolarization ability of *o*-orsellinaldehyde on primary microglial cells. Nevertheless, due to the absence of Arg-1 stimulation, cells could hypothetically have reached an intermediate phenotype closer to a M2 state.

## 4. Discussion

There is increasing evidence that neuroinflammation plays an essential role in the progression of neurodegenerative disorders [32]. More concisely, the aberrant activation or functional impairment of microglia/astrocytes in the brain exacerbates neuroinflammatory responses, leading to neurodegenerative pathologies [33]. In this regard, microglial activation has been identified as a hallmark of this inflammatory process [34,35]. Microglia cells, the resident macrophages of the central nervous system, perform a very active, continuous surveillance function. However, the long-term activation of microglia results in an excessive release of cytokines and inflammatory mediators (i.e., IL-1β, COX-2, TNF-α) that will ultimately have neurotoxic consequences [36]. Interestingly, despite microglia promoting neuroinflammation, they only represent around the 10% of the whole cellular population. In fact, astrocytes are the most abundant glia cell type of the central nervous system (CNS). Interestingly, this last cell type is essential for brain homeostasis, as it provides growth factors and metabolites to neurons. Additionally, astrocytes also support neuronal plasticity and synapse maturation, and regulate the extracellular balance of ions and neurotransmitters [37]. Nevertheless, activated astrocytes are also able to release a myriad of molecular signals that contribute to the inflammatory state of the brain after disease or injury through the direct activation of the immune defenses with the concomitant release of chemokines, cytokines, and other growth factors [38]. Therefore, the neuroinflammatory response modulation of the microglial/astrocytic function in the CNS could be a potentially interesting therapeutic approach for the prevention or treatment of neuroinflammation and neurodegeneration-related diseases and has been widely explored [39,40]. Besides drugs, natural products represent an interesting source of bioactive molecules [41]. In neuroinflammation, numerous studies have shown that the beneficial effects of natural compounds were induced thanks to their antiapoptotic, anti-inflammatory, antioxidant, and neuroprotective effects [42,43].

On the other hand, among the different natural sources of functional molecules, mushrooms contain a wide array of bioactive ingredients that encourage their application in disease prevention and human health maintenance [44].

Considering this context, our results show that the previously *in silico* identified natural compound *o*-orsellinaldehyde from the *G. frondosa* mushroom is able to potently counteract neuro-inflammation both in microglia and in astrocytes. Nevertheless, a general criticism regarding the potential effect of natural compounds on CNS is its poor or absent ability to cross BBB. Interestingly, the computational prediction shows that *o*-orsellinaldehyde, theoretically, is able to permeate BBB due to its physicochemical characteristics (shown in Supplementary Figure S1). Nevertheless, one clear limitation of the present research is that despite this BBB permeability prediction we will not be sure of the effectiveness on the BBB crossing until in vivo tests of the anti-neuroinflammatory properties of the studied compound have been done. Nevertheless, the fact that it could be able to effectively reach the CNS and its in vitro toxicity absence (shown in Figure 1 and Figure 2) represent hopeful/encouraging aspects for its future in vivo application. 

Interestingly, the application of mushroom-derived compounds to neuroinflammatory conditions is an active field of research and many beneficial species, both from culinary and traditional medicine fields, have been described [8] . For example, *Antrodia camphorata* was the first mushroom described to possess anti-neuroinflammatory activities [45]. The methanol extract of the wild fruiting bodies (MW) was shown to dose-dependently reduce both iNOS and TNF-α mRNA expression in LPS/IFN γ-activated EOC13.31 mouse microglial cells. In the same study, MW of *A. camphorata* successfully inhibited the phosphorylation of STAT, ERK, JNK, and the activation of NF-κB in the LPS/IFN γ-activated EOC13.31 microglial cells. The effectiveness of MW was further shown in β-amyloid-activated microglia in which iNOS and COX-2 protein expression were remarkably reduced [46]. Another interesting mushroom (*Ganoderma Lucidum*) showed promising anti-neuroinflammatory properties. In this case, the ethanolic extract of the *G. lucidum* fruiting bodies (EGL) was able to significantly reduce, in a dose-dependent manner, LPS-stimulated NO, PGE2, IL-1β and TNF-α production along with a transcriptional suppression of iNOS, IL-1β, TNF-α, and COX-2 in BV2 microglia cells [47]. Additionally, EGL was also able to prevent the degradation of IκBα, the NF-κB p65 subunit nuclear translocation as well as the NF-κB transcription in LPS-stimulated BV2 microglial cells. On the other hand, a significant inhibition on the protein expression of MyD88 and TLR4 was also induced by EGL. This fact suggested that the anti-neuroinflammatory properties of EGL involved both TLR4 and MyD88, as well as the downstream NF-κB pathway [47]. These examples point out that some important advantages of many natural compounds in neuroinflammation are related to their high affinity for various receptors in the brain, thereby specifically promoting or inhibiting various molecular signal transduction pathways and their multi-targeting effects on various CNS disorders as well as their lower side effects compared to conventional synthetic drugs [9,10,11,12,13,14].

As was previously seen, nitric oxide production and iNOS expression represent two important targets for inflammation studies in macrophage and glial cells. Three different NO synthases are responsible for the generation of Nitric oxide (NO) from L-arginine. Of these, two are constitutive isoforms and the third is an inducible and Ca^2+^-independent NO synthase (iNOS). Additionally, iNOS is expressed only following transcriptional activation of its gene [48]. NO plays an important role in neurotransmission, vascular function, host defense, and immune regulation [49]. Such transcriptional activation can be mediated in response to the gram-negative bacterial infections that can be experimentally simulated by treating the abovementioned cells with LPS. Interestingly, the *G. frondosa* mushroom identified compound (*o*-orsellinaldehyde) was able to effectively blunt both NO production (expressed as nitrite levels) and iNOS protein expression in astrocytes and microglia cells (Figure 3 and Figure 4). This is in line with the studies that have been previously introduced, where several mushroom extracts from other species are also able to decrease iNOS expression. Furthermore, aqueous and methanolic extracts of multiple mushroom species (e.g., *Hericium erinaceus*, *Pleurotus ostreatus,* and *Sarcodon scabrosus*) have been reported as having important anti-neuroinflammatory properties [50,51,52]. Nevertheless, few of the previous studies have clearly identified the molecule/s present in the extract that could be responsible for the anti-inflammatory effect. In our case, a previous research where virtual screening techniques against IKβα were used, identified one valuable molecule with potential anti-inflammatory properties. Subsequently, the identified molecule (*o*-orsellinaldehyde) was identified in the *G. frondosa* mushroom. In fact, this molecule had been previously identified as valuable compound by Lin and collaborators [53], when studying the anti-tumor activity of bioactive metabolites produced by submerged culture of *G. frondosa*. Interestingly, they discovered that the most active cytotoxic metabolite was *o*-orsellinaldehyde. Interestingly, several authors have also reported the anti-inflammatory properties of *G. frondosa* extracts [54,55,56]. Additionally, other authors have also reported the neuroprotective effects of *G. frondosa* polysaccharide [57,58]. Nevertheless, as far as we know, our research shows for the first time the neuro-anti-inflammatory and neuro-modulatory effects on astrocytes and microglia of a small molecule derived from this mushroom. One of the results that support this affirmation is the fact that *o*-orsellinaldehyde is able to downregulate, both in astrocytes and microglia, the LPS-induced overexpression of the hemoxigenase-1 (HO-1) enzyme (Figure 5).

HO is the limiting enzyme in heme degradation, and produces iron, biliverdin, and carbon monoxide. Subsequently, biliverdin reductase converts biliverdin to bilirubin, which is a powerful antioxidant molecule [59]. Furthermore, three isoforms have been characterized in mammals (HO-1, HO-2, and HO-3), being HO-1 the inducible one. The expression of HO-1 is stimulated by several stress factors such as proinflammatory cytokines, LPS, UV light, hypoxia, heat shock, heavy metals, and NO [60,61]. The increase on this enzyme is thought to trigger an adaptive mechanism that protect cells from oxidative injury [30]. Interestingly, HO-1 knockout mice present higher mortality rates and organ damage when treated with LPS than wild-type animals [62]. In the CNS, HO-1 is normally expressed at a very low level, but is quickly induced in some neurons, microglia and astrocytes, by extravascular hemoglobin, hemin, and a variety of oxidants [63,64]. On the other hand, HO-2 is expressed predominantly by neurons in a constitutive way [63].

Interestingly, our results show that the *G. frondosa*-derived molecule (*o*-orsellinaldehyde) dose-dependently inhibit the HO-1 expression in both glial cell populations. This fact points out the potential protective effect of the natural compound which is evidenced by the lack of HO-1 protective dependency despite LPS presence. Additionally, as the TLR-4 dependent overexpression of HO-1 by the endotoxin is intracellularly mediated by the phosphorylation of several kinases (IKβ kinases, protein kinase C, PI3 Kinase, and MAP kinases), which will in turn activate some transcription factors, as NF-κβ and AP-1 [65] (Saha S); the abrogation of HO-1 expression by *o*-orsellinaldehyde should also encompass some of the described upstream signaling steps. Effectively, as it can be seen in Figure 6, the natural compound is also able to inhibit IKβα, p38, and JNK phosphorylation in microglial cells. Interestingly, the strongest inhibitory effect is seen in JNK phosphorylation. In consequence, it could be stated that the anti-neuroinflammatory effect of the *G. frondosa* identified molecule is not only restricted to IKβα phosphorylation inhibition (NF-κβ pathway), and it is also participated by p38 and JNK phosphorylation inhibition (AP-1 pathway). This is an interesting and unexpected feature of this natural compound because it could promote a stronger anti-neuroinflammatory effect. Intriguingly, this fact could also imply that despite the virtual screening used for its identification was only restricted to the IKβα kinase target, the fact that it was predicted that *o*-orsellinaldehyde exerted a competitive activity in the ATP binding pocket of the IKβα kinase added to the fact that it is a small molecule, could be opening the possibility of an “illegitimate” effect on other kinases (p38 and JNK phosphorylation). Curiously, this fact has also been previously described in the literature both for BV2 and primary microglial cell cultures. For example, it has been shown [66] that the flavonoid Luteolin, which can be found in celery and green pepper, has a neuroprotective effect by inhibiting both NF-κβ and AP-1 pathways. On the other hand, it has been also shown that the 3-hydroxy-4-trifluoromethylbenzoic acid derivative named BECT, which is related to a fluorinated salicylate, is able to promote the inhibition of JNK, p38, and NF-κB signaling; and this might be one of the possible molecular mechanisms which contributes to the anti-neuroinflammatory effect of BECT in LPS-stimulated microglial BV2 cells [67]. Interestingly, it has also recently been shown that exosomes from adipose-derived stem cells alleviate neural injury caused by BV2 microglial activation via suppressing NF-κB and MAPK pathway [68].

Regarding the potential of *o*-orsellinaldehyde in promoting M2 polarization in LPS-activated microglia, we studied the expression of several genes associated with either M1 or M2 phenotypes. As Figure 7 shows, treatment with *o*-orsellinaldehyde induced a decrease in iNOS, IL-1β and TNF-α expression (M1 markers) and an increase in the mRNA expression of IL-10 and MRC-1 (M2 markers) in LPS-activated microglia in comparison to the control cultures. Unexpectedly, Arg-1, an M2-like marker, did not change its expression when activated microglia were treated with the tested compound. Nevertheless, this does not necessarily mean that *o*-orsellinaldehyde was unable to induce a glial polarization through an anti-inflammatory phenotype because the expression of Arg-1 was not increased. Classic M1/M2 classification is a simplification of matters and further intermediate phonotypes have been described [69]. Furthermore, it has also been reported that microglia express distinct M1 and M2 phenotypic markers in the postnatal and adult central nervous systems, and Arg-1 is one of these variable markers [70]. Interestingly, we clearly observed a change on cellular morphology between LPS treated primary microglia (ameboid shape) and LPS + *o*-orsellinaldehyde treated cells (ramified/stellate) cells. Taking all this into account, based on our results we can conclude that the tested molecule, *o*-orsellinaldehyde, is able to promote the microglia switch from the inflammatory M1 type to the anti-inflammatory M2 type. Interestingly, it has also been shown that the grapefruit flavonoid Naringenin by targeting the MAP kinase pathways is able to modulate microglia M1/M2 polarization in LPS-stimulated BV2 cultures [71]. Furthermore, anisomycin (which is a selective activator of JNK) abolished the Naringenin-induced M2 polarization and further Naringenin-inhibited BV2 activation. In this regard, an interesting possibility would be to further explore if the *o*-orsellinaldehyde M2 polarization of primary microglial cells is fully or partially dependent on the MAP kinase pathways. Altogether, these results postulate that the beneficial effects of the *G. frondosa* mushroom on the CNS could not be only due to its polysaccharide component but also to the presence of *o*-orsellinaldehyde that might be exerting some beneficial effects through its anti-inflammatory and immunomodulatory properties on glial cells. Nevertheless, additional mechanistic and preclinical studies are needed to ascertain its real potential use as anti-neuroinflammatory and neuroimmunomodulatory agent.

## Figures and Tables

**Table 1:** Detailed sequences of the primers used in the Q-PCR experiments.

Gene	Forward Primer (5′-3′)	Reverse Primer (5′-3′)	Accession Number	Size (bp)
iNOS	GCCACCTCGGATATCTCTTG	TCTGGGTCCTCTGGTCAAAC	NM_0126113	81
IL-1β	CACCTCTCAAGCAGAGCACAG	GGGTTCCATGGTGAAGTCAAC	M98820	79
IL-10	GCCAAGCCTTGTCAGAAATGA	TTTCTGGGGCCATGGTTCTCT	NM_012854.2	73
Arg-1	ATATCTGCCAAGGACATCGTG	AGGTCTCTTCCATCACTTTGC	NO_17134	141
MRC-1	TGGACTAAGCCAAGGGGCAA	CAGGAGCAGGGGGAGTCTCA	NM_001106123	121
HPRT	CTCATGGACTGATTATGGACAGGAC	GCAGGTCAGCAAAGAACTTATAGCC	S79292	123

## Data Availability

The data presented in this study are available on request from the corresponding author. The data are not publicly available due to lack of platform to publish it.

## Supplementary Materials

The following are available online at https://www.mdpi.com/article/10.3390/pharmaceutics13060806/s1, Figure S1. *o*-orsellinaldehyde is able to cross blood-brain barrier. The SwissADME web tool was used to predict the ADME parameters of the *o*-orsellinaldehyde molecule (right-side). Additionally, the BOILED-Egg method was used to predict blood-brain barrier (BBB) permeation (left-side). As can be seen in the image, due to its physicochemical properties, *o*-orsellinaldehyde is located in the “yolk” region of the picture (orange region), which indicates that, theoretically, it is able to permeate through the BBB. (source: http://www.swissadme.ch/index.php; accessed date 1 February 2021).
