# Supplementary material for: Anti-Inflammatory and Immunomodulatory Effects of the Grifola frondosa Natural Compound o-Orsellinaldehyde on LPS-Challenged Murine Primary Glial Cells. Roles of NF-κβ and MAPK
Source: Pharmaceutics. 2021 May 28;13(6):806. doi: 10.3390/pharmaceutics13060806 (PMC8229786; doi:10.3390/pharmaceutics13060806)
Supplement: Supplementary file 1 [file pharmaceutics-13-00806-s001.zip › pharmaceutics-1210434-supplementary.pdf]

pharmaceutics

MDPI

Supplementary Materials: Anti-Inflammatory and Immunomodulatory Effects of the *Grifola Frondosa* Natural Compound *o*-Orsellinaldehyde on LPS-Challenged Murine Primary Glial Cells. Roles of NF-Kb and MAPK.

Sarah Tomas-Hernandez, Jordi Blanco, Santiago Garcia-Vallvé, Gerard Pujadas, María José Ojeda-Montes, Aleix Gimeno, Lluís Arola, Luisa Minghetti, Raúl Beltrán-Debón and Miquel Mulero

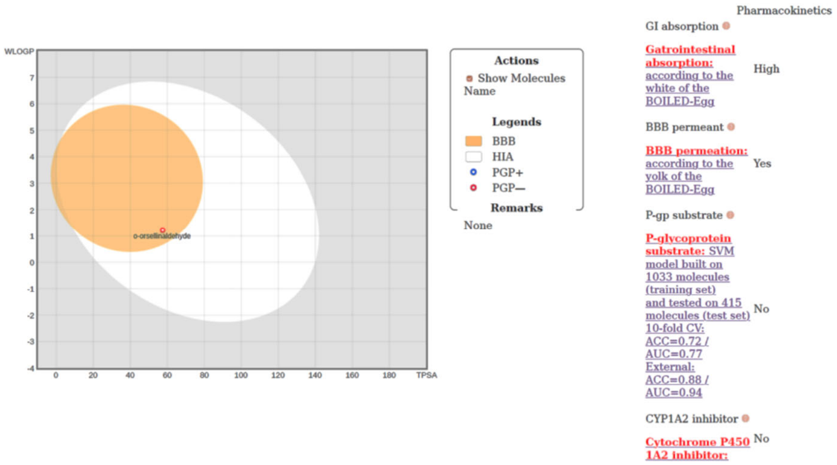

Figure S1. *o*-orsellinaldehyde is able to cross blood-brain barrier. The SwissADME web tool was used to predict the ADME parameters of the *o*-orsellinaldehyde molecule (right-side). Additionally, the BOILED-Egg method was used to predict blood-brain barrier (BBB) permeation (left-side). As can be seen in the image, due to its physicochemical properties, *o*-orsellinaldehyde is located in the "yolk" region of the picture (orange region), which indicates that, theoretically, it is able to permeate through the BBB. (source: http://www.swissadme.ch/index.php; accessed date 1/02/2021).
